# Metastatic Early-Onset Colon Cancer With BRCA2 Mutation Presenting With a Large Obstructing Pelvic Mass and Causing Acute Liver Failure and Acute Hypoxic Respiratory Failure

**DOI:** 10.7759/cureus.64770

**Published:** 2024-07-17

**Authors:** Sabastain F Forsah, Sini Bijoy, Divine Besong Arrey Agbor, Derek Ugwendum, Margarieta Okang, Uroosa M Syed, Cinthya Aviles, Jay Nfonoyim

**Affiliations:** 1 Internal Medicine, Richmond University Medical Center, Staten Island, USA; 2 Hematology and Oncology, Richmond University Medical Center, Staten Island, USA; 3 Pulmonary and Critical Care Medicine, Richmond University Medical Center, Staten Island, USA

**Keywords:** obstructive features, brca2, colorectal cancer, metastasis, acute liver failure, acute hypoxic respiratory failure, early-onset crc

## Abstract

Colorectal cancer (CRC) still constitutes a significant healthcare burden. Although its overall incidence is reducing, the incidence of early-onset CRC is increasing. There is uncertainty about the association between CRC and *BRCA2* mutations and also, even though most cancers metastasize to the liver, acute liver failure (ALF) from metastatic cancer and specifically CRC is uncommon. This is a case of a young patient with *BRCA2* mutation who presented with a large obstructing CRC with extensive metastatic burden to the liver, causing a fatal ALF. This case shows the growing number of ALFs associated with metastatic disease and suggests a possible association between *BRCA2* mutation and CRC. This association needs more research to establish.

## Introduction

Colorectal cancer (CRC) represents a major worldwide healthcare burden. It is the third most common cancer in the United States and worldwide in both males and females [1-3]. It is also the second most common cause of cancer-related deaths worldwide [2]. The overall incidence of CRC has decreased due to early detection through screening. However, the incidence of early-onset CRC, in people aged less than 50 years, has shown an increase. The reason for this increase is not known but there is the suggestion that it is related to lifestyle such as diet, physical activity, and obesity [3]. Due to this increase, the American Cancer Society and the United States Preventive Services Task Force lowered the age for starting screening colonoscopy in the general population from 50 years to 45 years [4]. 

Colon cancer is often sporadic with race, a diet rich in processed red meat, cigarette smoking, alcohol consumption, prior radiation, and the presence of inflammatory bowel disease being some risk factors [2]. Hereditary colon malignancies make up about 5% of cases and are most commonly associated with hereditary nonpolyposis colon cancer and genetic polyposis syndromes [2]. Although there is still no consensus on the association between BRCA1/2 mutations and CRC, there is the suggestion that *BRCA1* is associated with an increased risk of early-onset colon cancer but the association between CRC and *BRCA2* mutation remains uncertain [3].

CRC can be asymptomatic or can present with symptoms due to the local tumor or metastatic disease. About 20% of patients with CRC already have metastases at diagnosis [5]. Colon cancer mostly metastasizes to the liver followed by the lungs and metastasis to distant organs is the predominant basis for CRC mortality with a five-year survival rate of approximately 14% [6]. Acute liver failure (ALF) due to metastatic infiltration is rare even though the liver is one of the most common organs involved in metastatic disease [7]. This is a case of a young patient with *BRCA2* mutation who presented with metastatic colon cancer with a large pelvic mass which caused hydronephrosis and constipation. The cancer had a high burden of metastases to the liver causing ALF. It also metastasized to the lungs causing acute hypoxic respiratory failure.

This case has two important clinical implications. Firstly, it emphasizes that, though not very common, metastatic disease can cause overt ALF if the metastatic burden to the liver is high. It also suggests that *BRCA2* mutation can increase the risk of developing rapidly growing, aggressive, and early-onset colon cancer.

## Case presentation

A 44-year-old male with a recent diagnosis of colon adenocarcinoma presented to the emergency room (ER) with worsening abdominal pain, abdominal distention, and jaundice. His symptoms started about two months prior to presentation with abdominal pain, abdominal fullness, decreased oral intake, constipation, rectal pain, and weight loss for which abdominal imaging revealed a large pelvic mass. Colonoscopy with biopsy showed an adenocarcinoma of the colon. The patient had a family history of ovarian cancer in the mother and prostate cancer in the grandfather. The patient smoked half a pack of cigarettes per day for 10 years but quit about 10 years ago. He denied alcohol abuse, illegal drug use, and any other chronic liver pathology.

Vital signs on arrival showed a blood pressure of 101/60 mmHg, heart rate of 111/minute, respiratory rate of 18/minute, temperature of 98.1°F, and an oxygen saturation of 89% on room air. Physical examination revealed an alert and oriented patient with mild respiratory distress. He was lethargic and had frank jaundice, scleral icterus, abdominal distention, and generalized abdominal tenderness. There was hepatomegaly and a large mass extending from the right lower quadrant, crossing the abdominal midline to the left upper quadrant.

Initial laboratory investigations are shown in Table 1. Urine drug screen was negative, acetaminophen level was 12.6 ug/ml, and acetylsalicylic acid level was less than 3 ug/ml. Viral hepatitis panel and autoimmune hepatitis panel were negative. Though the white count was elevated, urinalysis and blood cultures were negative and he was afebrile. Model for End-Stage Liver Disease (MELD)-sodium (MELD-Na) score was 38 but he was not a liver transplant candidate due to his malignancy.

**Table 1:** Initial Laboratory Results ALP: Alkaline Phosphatase, ALT: Alanine Transaminase, AST: Aspartate Aminotransferase, BUN: Blood Urea Nitrogen, INR: International Normalized Ratio, PTT: Partial Thromboplastin Time

Laboratory parameters	Patient Numbers	Reference Range/units
Hematology		
White cell count	14900	4500–11,000/mm^3^
Hemoglobin	14.4	Male: 13.5–17.5 g/dL
Platelets	140	150,000–400,000/mm^3^
Biochemistry		
Sodium	116	136–146 mEq/L
Potassium	6.4	3.5–5.0 mEq/L
BUN	44	7–18 mg/dL
Creatine	1.57	0.6–1.2 mg/dL
AST	2170	12–38 U/L
ALT	767	10–40 U/L
ALP	532	25–100 U/L
Total bilirubin	22.2	0.1–1.0 mg/dL
Direct bilirubin	16.2	0.0–0.3 mg/dL
Lactic acid	10.1	0.5 – 2.2 mmol/L
Lipase	36	12 – 53 U/L
Ammonia	84	11 – 32 umol/L
Carcinoembryonic antigen	143	0 – 2.9 ng/mL
Coagulation factors		
PTT	45.7	25–40 seconds
INR	2.44	0.9-1.12 seconds
Fibrinogen	215	200-400 mg/dL

**Figure 1:**
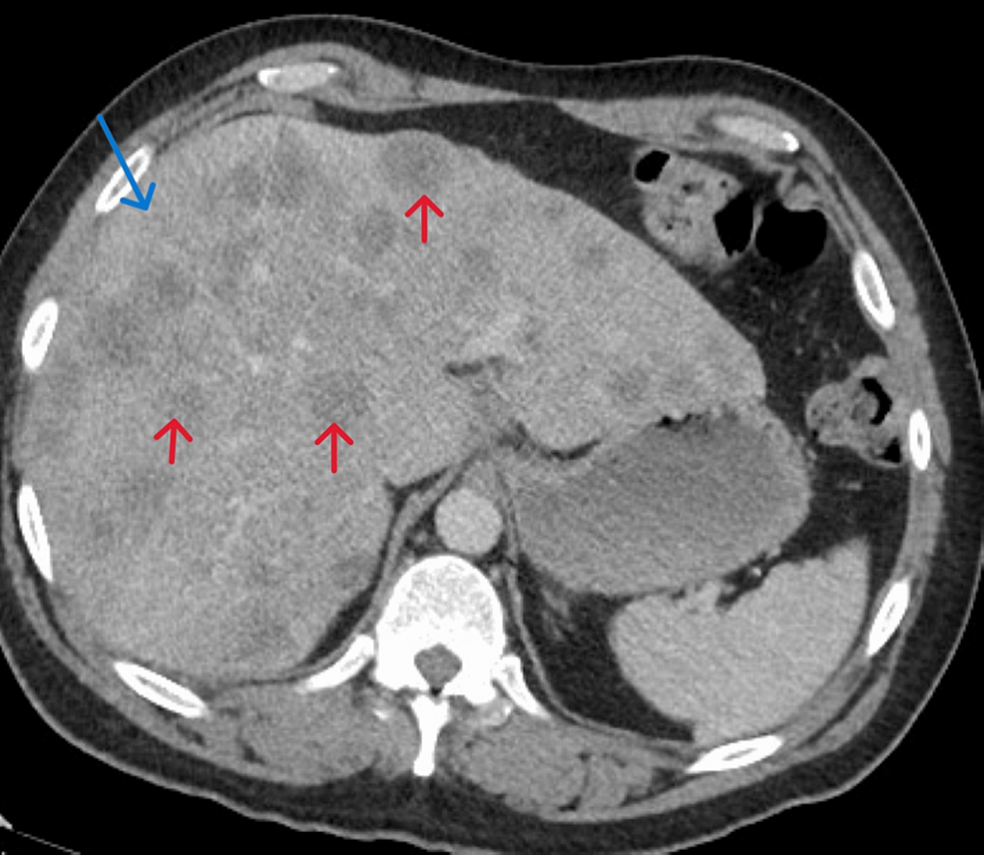
CT of the abdomen revealing a massive hepatomegaly (blue arrow) with innumerable hypodensities (red arrows) scattered throughout its parenchyma

**Figure 2:**
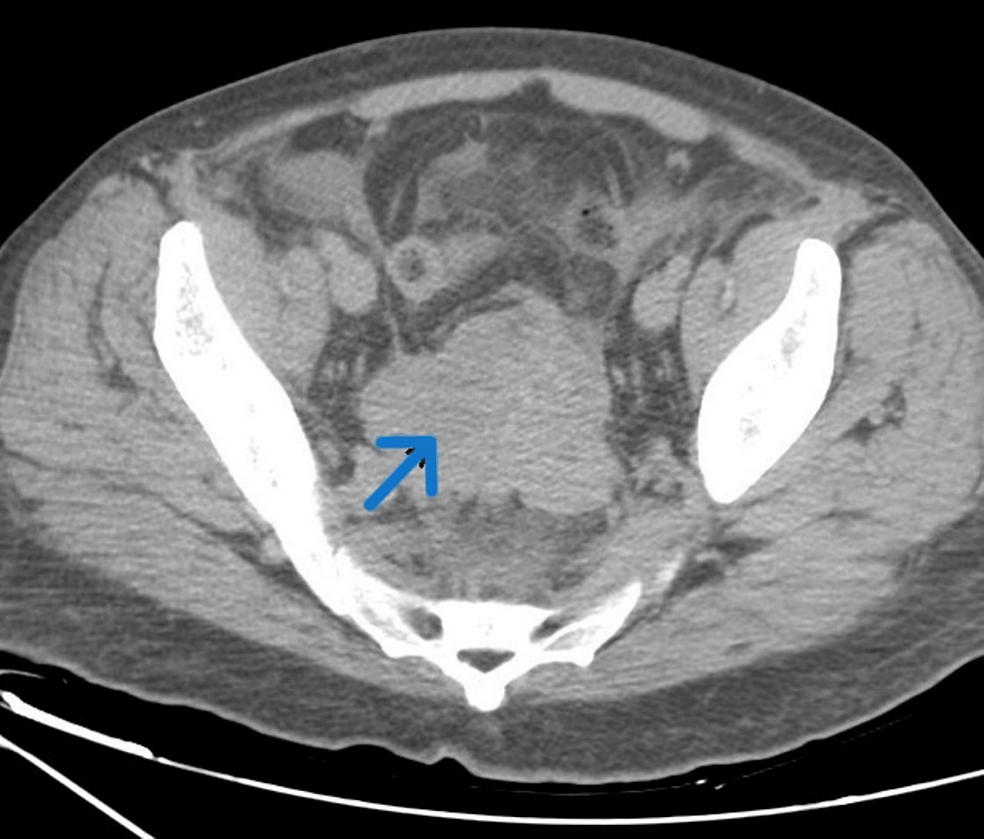
CT of the pelvis showing a large soft tissue mass in the pelvis

**Figure 3:**
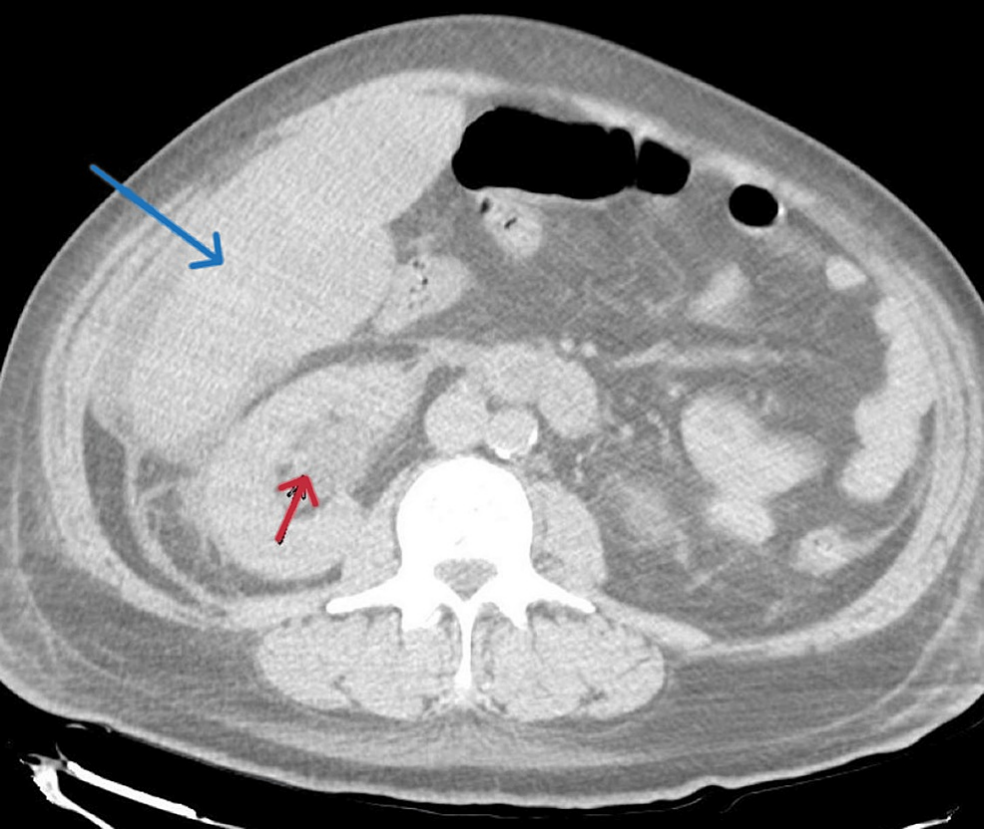
CT abdomen and pelvis showing a right-sided hydronephrosis (red arrow) due to a large pelvic mass encasing the right ureter. The blue arrow shows the liver

Computed tomography (CT) of the abdomen and pelvis revealed a massive hepatomegaly with innumerable cysts scattered throughout its parenchyma nearly completely replacing the normal hepatic tissue (Figure 1). There was a soft tissue mass in the pelvis (Figure 2) extending to the retroperitoneum and encasing the distal ureter causing moderate right-sided hydronephrosis (Figure 3).

**Figure 4:**
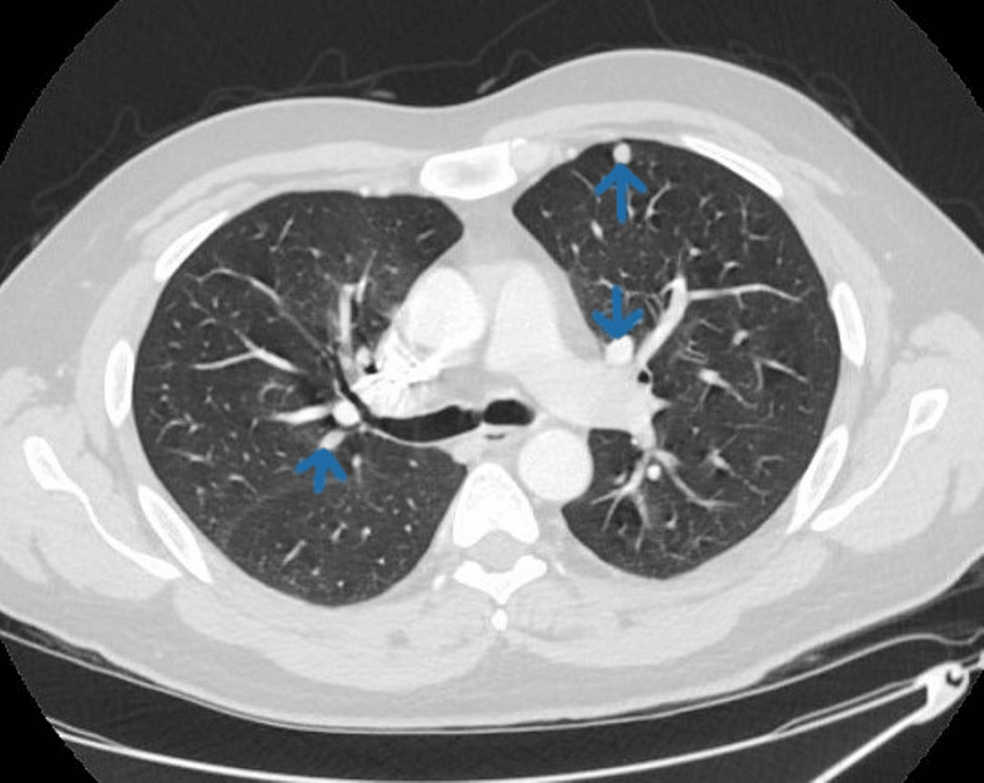
CT of the chest showing multiple bilateral nodular lesions (blue arrows) in the lungs

Magnetic resonance cholangiopancreatography (MRCP) revealed hepatomegaly with no dilation of intrahepatic and extrahepatic biliary ducts. CT chest revealed multiple bilateral soft tissue densities throughout the lung parenchyma (Figure 4).

**Figure 5:**
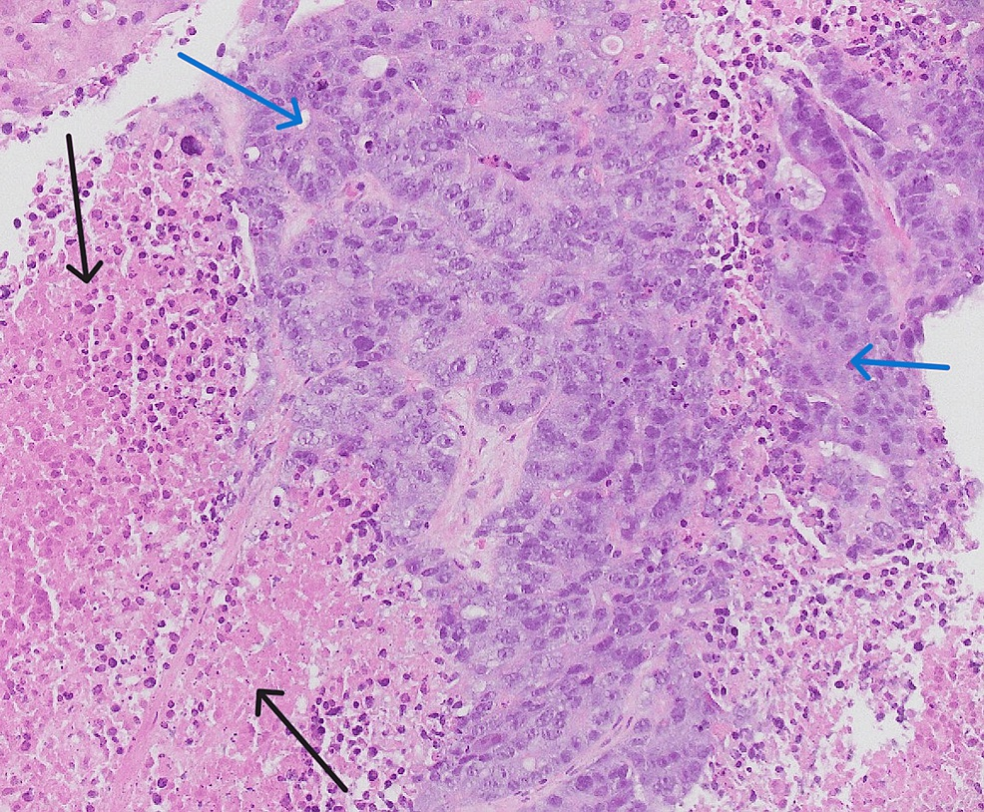
Liver biopsy showing minimal viable liver tissue; Blue arrows show metastatic disease while black arrows show necrotic liver tissue.

Liver biopsy results showed metastatic colon adenocarcinoma with extensive necrosis (Figure 5). Next-generation sequencing (NGS) of the mass revealed* BRCA2, APC*, and *TP53* mutations while the NGS of blood revealed high microsatellite instability (MSI), *BRCA2* 91%, and *APC* 72% in addition to *TP53*, *CDK8*, *EGFR*, *CDK4*, *CCNE1* genes. Genetic testing showed heterozygosity for *BRCA2*, *BARD1*, *MBD4* genes.

The patient was admitted to the medical intensive care unit and the six-day hospital course was marked by worsening respiratory failure with increased supplemental oxygen needs which culminated in him being intubated on day 5 of admission. He also became hypotensive requiring initiation of vasopressors and empirical antibiotics. His renal function worsened and he became anuric. See Table 2 for trends of some laboratory findings during the course of hospitalization. The patient was initially scheduled to start pembrolizumab, an immune checkpoint inhibitor, due to the high MSI found in the tumor, but he became hemodynamically unstable, progressed to multiorgan failure, and later passed away on day 7 of hospitalization.

**Table 2:** Trends of selected laboratory investigations; Day 0 is day of admission ALP: Alkaline Phosphatase, ALT: Alanine Transaminase, AST: Aspartate Aminotransferase, BUN: Blood Urea Nitrogen, Cr: Creatinine, D. bil: Direct Bilirubin, INR: International Normalized Ratio, K: Potassium, phos: Phosphorus, PTT: Partial Thromboplastin time, T.bil: Total Bilirubin

	Unit	Reference	Day 0	Day 1	Day 2	Day 3	Day 4	Day 5	Day 6
AST	U/L	<34	2170	3740	4782	4276	4529	3772	4211
ALT	U/L	10-49	767	1288	1230	1223	1203	1094	1169
ALP	U/L	46-116	532	488	491	510	475	518	543
T.bil	mg/dL	0.2-1.0	22.2	22.8	23.3	26.5	22.9	22.5	20.9
D.bil	mg/dL	0.0-0.3	16.2	16.4	16.7	16.8	17.8	17.1	14.6
Cr	mg/dL	0.70-1.30	1.57	2.04	2.47	3.17	3.85	4.12	4.23
BUN	mg/dL	7–18	44	56	59	64	62	66	72
INR	sec	0.9-1.12	2.44	2.49	2.87	2.70	3.44	2.56	3.46

## Discussion

ALF is the development of severe liver injury with encephalopathy and coagulopathy (international normalized ratio (INR) > 1.5) in the absence of pre-existing liver disease, with an illness of <26 weeks duration [8]. The most common causes of ALF are acetaminophen overdose, idiosyncratic drug reactions, viral hepatitis, and autoimmune hepatitis [9]. Malignant infiltration of the liver is a rare cause of ALF, despite the liver being the most common location for the spread of tumors, with the reason not well known. Primary tumors involved can either be solid organ tumors or hematological malignancies [7-10]. In a study carried out in 1998 in London covering an 18-year period, only 18 patients (0.44%) had ALF caused by malignant infiltration of the liver [10]. Another study in 2014 examined a large, multi-center ALF registry over a 15-year period from 1998 to 2012 [8]. Among the 1910 patients with ALF, only 27 (1.4%) were attributed to malignant infiltration of the liver. The common malignancies involved included lymphoma or leukemia (33%) and breast cancer (30%), with colon cancer making up only 7% of the cases [8]. Since then, few other case reports have been written about malignant infiltration as a cause of ALF but data on metastatic colon cancer causing ALF are scarce [7,9]. 

Metastasis is the process by which cancer cells spread from the primary tumor to distant organs. This process involves many steps which include detachment from primary cancer, invasion of surrounding tissues, entry into lymphatic or vascular systems, avoidance of host defense mechanisms, arrest at a new site, extravasation into the tissue, and growth at the new site [11,12]. The metastatic process involves the interaction between many tumor and patient factors, and these determine the site of tumor spread. These factors include the origin of the tumor, intrinsic properties of the tumor, the patient's tissue affinities, and circulation patterns [11]. CRC likely spreads by draining via the portal vein into the liver. The liver is made up of fenestrated endothelial cells forming the sinusoidal endothelial layer which lacks a basement membrane, making it easy for circulating cells to have access to the liver tissues [11]. The lungs are the second most common site of CRC spread [6]. From the liver, cancer cells spread to the lungs, via the heart. Also, lymphatic drainage from the gastrointestinal system drains through cisterna chyli to the left subclavian vein, and to the lungs [5]. 

Metastatic colon cancer with metastases to the liver can present with abdominal pain, ascites, jaundice, hepatic encephalopathy, splenomegaly, and hepatomegaly like in our patient, and depending on the size and location of the mass, it can also cause obstructive features [6]. 

Like in our patient, laboratory investigations in ALF caused by metastatic liver disease show a mixed hepatocellular/cholestatic picture with prolonged INR and low platelet counts. Bilirubin and aminotransferases are very elevated, with values above 40 times the upper limit of normal [8]. Diagnosis of metastatic disease is done by imaging studies but sometimes imaging studies fail to reveal malignant liver infiltration, which is seen postmortem [7]. The gold standard for diagnosis of metastatic liver disease is through liver biopsy. Biopsy may show a plug of metastatic carcinoma that is filling and obstructing a sinusoid. It can also reveal acute hemorrhagic necrosis consisting of hepatocyte debris and blood. This pattern of necrosis is a result of acute interference of incoming blood flow from a portal venule or hepatic artery embolized by metastatic cancer [7,8]. Our patient’s biopsy result shared similar features.

Chemotherapy has improved the survival of patients with metastatic liver disease but it is challenging to initiate such treatment on patients like ours who are unstable and severely ill with multi-organ failure. Complications of ALF include encephalopathy, cerebral edema, sepsis, renal failure, and respiratory failure [7]. Although our patient was afebrile with negative cultures, he was treated empirically with broad-spectrum antibiotics in the setting of high white blood cell count and hypotension, due to the high risk of developing sepsis in these patients. Approximately 56% of patients with CRC die from the cancer [6]. In the 2014 study involving patients with ALF from malignant infiltration of the liver, mortality was 85% within three weeks of hospitalization [8]. Our patient died after six days of hospitalization probably because he arrived when the disease was too advanced with extensive metastasis and liver failure.

*BRCA* is a tumor suppressor gene involved in DNA repair, cell proliferation control, and apoptosis. *BRCA1* and *BRCA2* mutations are the pathogenic variants of *BRCA* that cause loss of function in BRCA1/2 genes and result in genomic instability and tumor development [13], which might have been the case in our patient. The *BRCA2* pathogenic variant has been associated with breast, ovarian, prostate, and pancreatic cancers, and melanoma. However, studies about its association with CRC cancer show mixed results [3]. Some studies suggested that BRCA1/2 mutations might determine an increase in CRC diagnosis, particularly in young patients [14,15] while others did not observe any statistically significant difference in terms of CRC risk among *BRCA* mutation carriers [16,17]. Yet, others highlighted that only *BRCA1* carriers were at a higher risk of developing CRC [18]. Our patient had a genomic instability-causing *BRCA2* mutation which might have played a crucial role in increasing the risk for the development of colon cancer in this young patient. While there is no consensus on the impact of germline *BRCA2* mutations on CRC risk, a series of studies have shown that, in patients with CRC, *BRCA* mutations might increase the likelihood of response to oxaliplatin-based chemotherapy [3]. 

Medical personnel should consider metastatic infiltration of the liver as a differential diagnosis of ALF in a patient with metastatic cancer. Also, more research is needed to ascertain the relationship between *BRCA2* mutations and the possible increased risk of developing CRC, as seen in our patient.

## Conclusions

CRC continues to be a substantial public health problem; however, continuous research and progress in early detection and treatment provide optimism for the future. Further research into therapeutic options is still needed to increase survival in patients with ALF due to metastatic liver disease. More research is also needed to clearly establish the association between *BRCA2* mutation and the development of early-onset and aggressive colon cancer and to inform decisions on when colon cancer screening should be initiated in patients with *BRCA2* mutation if an association is found. Overall, patient awareness and adherence to screening recommendations will reduce the morbidity and mortality associated with CRC.

## References

[1] Spaander MC, Zauber AG, Syngal S, Blaser MJ, Sung JJ, You YN, Kuipers EJ (2023). Young-onset colorectal cancer. Nat Rev Dis Primers.

[2] Menon G, Recio-Boiles A, Lotfollahzadeh S (2024). Colon cancer. StatPearls [Internet].

[3] Maccaroni E, Giampieri R, Lenci E (2021). BRCA mutations and gastrointestinal cancers: when to expect the unexpected?. World J Clin Oncol.

[4] Tanaka LF, Figueroa SH, Popova V, Klug SJ, Buttmann-Schweiger N (2023). The rising incidence of early-onset colorectal cancer. Dtsch Arztebl Int.

[5] Riihimäki M, Hemminki A, Sundquist J, Hemminki K (2016). Patterns of metastasis in colon and rectal cancer. Sci Rep.

[6] Shin AE, Giancotti FG, Rustgi AK (2023). Metastatic colorectal cancer: mechanisms and emerging therapeutics. Trends Pharmacol Sci.

[7] Weeraddana P, Thomas T, Weerasooriya N, Mohamed Jiffry MZ, Golovyan D, Bal S (2022). A rare case of acute liver failure secondary to miliary liver metastasis. Cureus.

[8] Rich NE, Sanders C, Hughes RS (2015). Malignant infiltration of the liver presenting as acute liver failure. Clin Gastroenterol Hepatol.

[9] Kubo K, Kimura N, Mabe K, Matsuda S, Tsuda M, Kato M (2020). Acute liver failure associated with diffuse large B-cell lymphoma: an autopsy case report. Clin J Gastroenterol.

[10] Rowbotham D, Wendon J, Williams R (1998). Acute liver failure secondary to hepatic infiltration: a single centre experience of 18 cases. Gut.

[11] Jin K, Gao W, Lu Y, Lan H, Teng L, Cao F (2012). Mechanisms regulating colorectal cancer cell metastasis into liver (review). Oncol Lett.

[12] Chambers AF, Groom AC, MacDonald IC (2002). Dissemination and growth of cancer cells in metastatic sites. Nat Rev Cancer.

[13] Moynahan ME, Pierce AJ, Jasin M (2001). BRCA2 is required for homology-directed repair of chromosomal breaks. Mol Cell.

[14] Mauri G, Sartore-Bianchi A, Russo AG, Marsoni S, Bardelli A, Siena S (2019). Early-onset colorectal cancer in young individuals. Mol Oncol.

[15] Sopik V, Phelan C, Cybulski C, Narod SA (2015). BRCA1 and BRCA2 mutations and the risk for colorectal cancer. Clin Genet.

[16] Cullinane CM, Creavin B, O'Connell EP, Kelly L, O'Sullivan MJ, Corrigan MA, Redmond HP (2020). Risk of colorectal cancer associated with BRCA1 and/or BRCA2 mutation carriers: systematic review and meta-analysis. Br J Surg.

[17] Phelan CM, Iqbal J, Lynch HT (2014). Incidence of colorectal cancer in BRCA1 and BRCA2 mutation carriers: results from a follow-up study. Br J Cancer.

[18] Oh M, McBride A, Yun S (2018). BRCA1 and BRCA2 gene mutations and colorectal cancer risk: systematic review and meta-analysis. J Natl Cancer Inst.

